# Charge Transport Characteristics of Molecular Electronic Junctions Studied by Transition Voltage Spectroscopy

**DOI:** 10.3390/ma15030774

**Published:** 2022-01-20

**Authors:** Youngsang Kim, Kyungjin Im, Hyunwook Song

**Affiliations:** 1 Lawrence Berkeley National Laboratory, Berkeley, CA 94720, USA; kimys0916@gmail.com; 2 Department of Applied Physics, Kyung Hee University, Yongin 17104, Korea; limkj0512@khu.ac.kr

**Keywords:** molecular electronics, transition voltage spectroscopy, charge transport, single-level model

## Abstract

The field of molecular electronics is prompted by tremendous opportunities for using a single-molecule and molecular monolayers as active components in integrated circuits. Until now, a wide range of molecular devices exhibiting characteristic functions, such as diodes, transistors, switches, and memory, have been demonstrated. However, a full understanding of the crucial factors that affect charge transport through molecular electronic junctions should yet be accomplished. Remarkably, recent advances in transition voltage spectroscopy (TVS) elucidate that it can provide key quantities for probing the transport characteristics of the junctions, including, for example, the position of the frontier molecular orbital energy relative to the electrode Fermi level and the strength of the molecule–electrode interactions. These parameters are known to be highly associated with charge transport behaviors in molecular systems and can then be used in the design of molecule-based devices with rationally tuned electronic properties. This article highlights the fundamental principle of TVS and then demonstrates its major applications to study the charge transport properties of molecular electronic junctions.

## 1. Introduction

New development of nanoscale devices utilizing individual molecules with active functional elements is a promising approach towards the ultimate miniaturized electronics [1,2,3,4,5,6,7]. It is highly significant to understand and then manipulate the transport characteristics of carrying charges across molecular junctions, key for the construction of a single-molecule device [8,9,10,11,12,13]. For molecule-based devices, a complete understanding of the relationship between the carrier transport and the electronic structure is necessary to create devices with prescribed electronic functions [14,15,16,17,18,19,20,21,22,23,24,25]. A conventional density functional theory (DFT) calculation has been employed to estimate the energy level alignment, molecular conformation, and contact topology in the junctions [10,14,26,27,28,29]. However, these calculations cannot commonly be fulfilled by nonexperts. On the contrary, the Simmons model based on a simple rectangular barrier picture seems not to tender a reasonable description for the field of molecular electronics [30,31,32,33], mostly because it is difficult to illustrate the intrinsic chemical nature of constituent molecules. Recent works by diverse research groups have consistently shown that transition voltage spectroscopy (TVS) [34,35,36,37,38,39,40,41,42], especially on the basis for the Landauer picture, can be a more accurate analytical tool, in which quantitative fits into experimental current (*I*)–voltage (*V*) curves allow conveniently extracting charge transport parameters, for instance, the alignment of the highest occupied molecular orbital (HOMO) or lowest unoccupied molecular orbital (LUMO), the tunneling transmission, and the coupling strength of molecule–electrode contacts.

TVS has been proposed to obtain information as to the energy difference (*ε_h_* or *ε*_l_) between the closest molecular orbital (HOMO or LUMO) and electrode’s Fermi level (*E*_F_) [31], which has for a long while been known as a key quantity of current–carrying molecular junctions [1,4,10]. Within the early proposal depending on the physics of field emission, transition voltage (*V_t_*) has approximated the applied bias by which the tunneling barrier is tilted from trapezoidal to triangular shape [31,32]. Here, *V_t_* indicated the height of the tunneling barrier. If *ε_h_* or *ε*_l_ is much greater than the broadening of molecular orbital levels thanks to the couplings with metallic electrodes, TVS can be reinterpreted by using a coherent transport model with one molecular level [34,43,44,45,46,47,48], providing an excellent quantitative description of the transport experiments for molecular junctions. In this Review, we demonstrate fundamental principles of TVS (Section 2), focusing on key working equations and then discuss its recent application to investigating charge carrier transport through molecular electronic junctions (Section 3).

## 2. Fundamentals of Transition Voltage Spectroscopy

### 2.1. Initial Tunneling Barrier Conjecture

**Figure 1:**
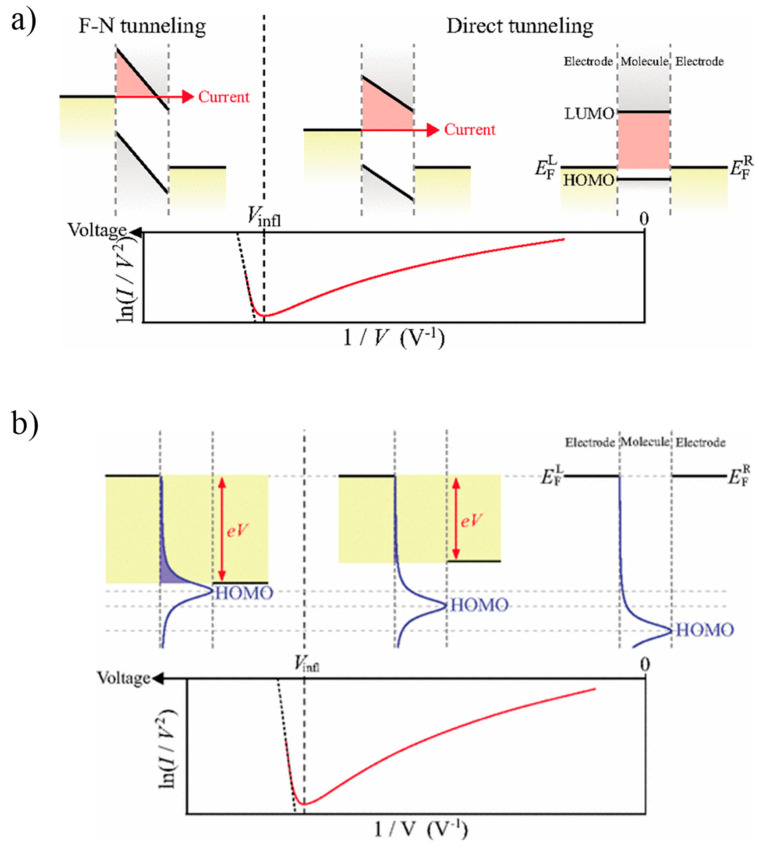
(**a**) Illustration of the Simmons tunneling model to show the inflection of FN curve. Triangle, trapezoidal, or rectangle in the upper panel represents the tunneling barrier with increasing the applied bias. (**b**) Illustration of the coherent molecular transport model based on a single level (HOMO) to show the inflection of FN curve. Reproduced with permission from [48].

By adopting the theoretical framework of Fowler-Nordheim tunneling (also well-known as field emission), Beebe et al. demonstrated that the position of the closest frontier molecular orbital (HOMO or LUMO) relative to *E*_F_ of the contacts can be estimated from *I*–*V* characteristics in two-terminal molecular junctions [31,32]. Initially, the TVS technique was simplified by a tunneling barrier picture using the Simmons model [49]. An inflection behavior in the Fowler-Nordheim (F–N) curves indicates a transition from direct tunneling (via a trapezoidal barrier) to field emission (via a triangular barrier) as described in Figure 1a. *V_t_* corresponds to the minimum point in the F–N plot that is *ln*(*I*/*V*^2^) against 1/*V* converted from *I*–*V* characteristics. By measuring *V_t_*, one can determine the tunneling barrier height (*Φ_B_*) that is equal to the energy interval of the *E*_F_ and the nearest molecular orbital level in charge of transport through the junctions.

In the zero-bias regime, the tunneling current through a rectangular barrier is approximated by

(1) $$I\propto V\exp(-2d\sqrt{2m_{e}\Phi_{B}}/\hbar )$$

where *m_e_* is the effective mass of electron, *d* is the width of tunneling barrier (typically equal to the length of the component molecules), and h=(2πℏ) is Planck’s constant. At the opposite regime, where the applied bias is greater than the potential barrier, the tunneling barrier starts thinning at *E*_F_ and is transformed from a trapezoidal shape to a triangular shape. Then, the *I*–*V* relationship is expressed as follows:

(2) $$I\propto V^{2}\exp(-4d\sqrt{2m_{e}\Phi_{B}^{3}}/3\hbar qV)$$

in which *q* is an electronic charge. For comparison, it is beneficial to linearize Equations (1) and (2) into logarithmic scale. Regarding the low-bias limit that direct tunneling occurs, Equation (1) is expressed as:

(3) $$\ln\left(\frac{I}{V^{2}}\right)\propto \ln\left(\frac{1}{V}\right)-\frac{2d\sqrt{2m_{e}\Phi_{B}}}{\hbar }$$

Similarly, for the high-bias regime resulting in field emission, Equation (3) scales as:

(4) $$\ln\left(\frac{I}{V^{2}}\right)\propto -\frac{4d\sqrt{2m_{e}\Phi_{B}^{3}}}{3\hbar q}\left(\frac{1}{V}\right)$$

From Equation (3), the F–N plot (that is $\ln\left(I/V^{2}\right)$ against (1/V) indicates a logarithmic increase, whereas it appears to be linear from Equation (4). Thus, a mechanistic change from direct tunneling to field emission displays a definite minimum bias voltage *V_t_*, indicating an inflection in the F–N plot (Figure 1a). This construction agrees with a change in the tunneling barrier with an increasing bias, starting from rectangular (zero bias) to trapezoidal (low bias) and then to triangular form (high bias). Thus, measurements of *V_t_* can provide a way to experimentally evaluate the height of the original rectangular barrier in molecular junctions. For a hole transport (p-type), the closest frontier molecular orbital level is the HOMO (thus, $\Phi_{B}=\left|E_{F}-E_{HOMO}\right|$), whereas for electron transport (n-type), it is the LUMO (thus, $\Phi_{B}=\left|E_{F}-E_{LUMO}\right|$).

Beebe et al. found from various π-conjugated thiols that *V_t_* was linearly scaled with $\Phi_{B}=\left|E_{F}-E_{HOMO}\right|$ [32]. On the contrary, for alkanethiol molecular junctions having different molecular lengths, *V_t_* was constant, which agrees with the fact that the energy level position of the nonconjugated alkyl molecules is independent of molecular lengths [32,50]. These results demonstrate a possibility that TVS can be employed as a spectroscopic technique to investigate electronic structures in a given molecular junction.

### 2.2. Coherent Molecular Transport Model

TVS has rapidly become one of the fascinating methods to probe molecular electronic junctions since its invention. However, because of the excessive simplicity of the Simmons tunneling model, the presented *I*–*V* data of molecular charge transport have been far from a full understanding. In order to gain a better interpretation of TVS, Huisman et al. employed a coherent tunneling transport model [43]. Within this transport model, the molecular energy levels are broadened in a Lorentzian shape by coupling with the contacts, leading to reasonable agreements with the molecular charge transport experiments. Furthermore, Araidai et al. demonstrated the inflection behavior in F–N plots with transmission functions for resonant peaks [48]. The presented calculation showed that such inflection does not essentially refer to the transition in the two tunneling regimes, direct tunneling, and field emission. By analyzing the relation of the inflection behavior in the F–N plots and transmission functions, the inflection could occur as the nearest molecular energy level was fairly approached to the resonant position within the bias window (Figure 1b). Even though the occasion of the inflection significantly is dissimilar to the conventional Simmons model, the F–N curves presented from respective calculations appear to be very analogous to those obtained from the latest experiments [37,39].

Within the coherent tunneling approach, the charge transport across the junctions is interpreted by the transmission function *T*(*E*) depending on the energy. *T*(*E*) is peaked in the region of the molecular orbital levels. It has been well described that a Lorentzian provides a reasonable approximation for *T*(*E*) around a single energy level as follows [44]:

(5) $$Τ\left(Ε;\varepsilon_{0},\Gamma \right)=\frac{f}{{\left(Ε-\varepsilon_{0}\right)}^{2}+\frac{\Gamma^{2}}{4}}$$

where $\varepsilon_{0}$ is the nearest frontier molecular orbital energy and Γ is a total of the broaden energy resulting from the coupling to both contacts. f is a constant factor to reflect the number of molecules existing in the junctions and the asymmetricity of coupling to both contacts. This approximation is independent of the actual value of f. In the range of finite voltage, the energy level is shifted in the respect to the position of zero voltage, as illustrated in Figure 1b. Considering the degree of contact asymmetry with a parameter η∈[−1/2;1/2], the *I*–*V* dependence can be estimated using the Landauer formula as follows [44,45]:

(6) $$I=\frac{2e}{h}\int_{-\infty }^{\infty }T\left(E;\varepsilon_{0}+\eta V,\Gamma \right)\left[f_{L}\left(V/2\right)-f_{R}\left(-V/2\right)\right]dE$$

where $f_{L/R}\left(V\right)=1/\left[\exp(E_{F}+eV\right)/k_{B}T+1]$ is the Fermi distribution functions for the left and right contact. For symmetric junctions η=0, and full asymmetric junctions have η=±1/2. By calculating Equation (6), one can find the minimum value of $\ln\left(I/V^{2}\right)$ at *V_t_*, where the slope of F–N curves is dependent quadratically on the applied voltage and the differential term $d\ln\left(I/V^{2}\right)/dV$ *d*[*ln*(*I*/*V*^2^)]/*dV* should be equal to zero.

### 2.3. Single-Level Model Analysis

Recent noteworthy works have described that the analytic single-level model (SLM) or Newns-Anderson Model, basically derived from the Landauer transport theory, can be employed to extract the effective energy offset (e.g., $\varepsilon_{h}=\left|E_{F}-E_{HOMO}\right|$ for p-type transport and $\varepsilon_{l}=\left|E_{F}-E_{LUMO}\right|$ for n-type transport) and the contact coupling *Γ* between molecules and electrodes from *I*–*V* characteristics of the junctions [34,51,52,53,54,55]. The excellent fits of the SLM to *I*–*V* curves have shown its validity to examine coherent, non-resonant tunneling transport that is typical of a great part of both aromatic and aliphatic junctions.

As long as the energy difference *ε_h_* (assuming p-type transport) is significantly greater than the broadening of energy levels arising from the molecule–electrode couplings, the interrelation between *V_t_* and *ε_h_* is described with regard to symmetric molecular junctions (that is, $\left|V_{t^{-}}\right|\approx V_{t^{+}}\equiv V_{t}$) as follows [34,41,45]:

(7) $$eV_{t}=2\varepsilon_{h}/\sqrt{3}$$

and then the *I–V* relationship are expressed as follows:

(8) $$I=GV\frac{\varepsilon_{h}^{2}}{\varepsilon_{h}^{2}-{\left(eV/2\right)}^{2}}$$

The conductance at zero-bias G=1/R can be given as follows:

(9) $$G=NG_{0}\frac{\Gamma^{2}}{\varepsilon_{h}^{2}}$$

where $\Gamma =\sqrt{\Gamma_{1}\Gamma_{2}}=\varepsilon_{h}\sqrt{G/NG_{0}}$ indicates the average interfacial coupling, *Γ*_1_ and *Γ*_2_ are decided by the energy level couplings to both electrodes (e.g., $\Gamma_{1}\approx \Gamma_{2}$ for symmetric cases), $G_{0}=2e^{2}/h$ is the conductance of quantum, and *N* is the total number of molecules contributing to the junction current.

Regarding the asymmetric molecular junctions (in the case of $\left|V_{t^{-}}\right|\neq V_{t^{+}}$), the molecular orbital energy *E_HOMO_*(*V*) (for p-type junctions) in a biased condition (V≠0) can be moved, relative to its position in the zero- bias $E_{HOMO}\equiv E_{HOMO}\;\left(V=0\right)$ by means of a quantity proportional to *V* [42,55].

(10) $$E_{HOMO}\left(V\right)=E_{HOMO}+\gamma eV,\;\varepsilon_{h}\left(V\right)=E_{F}-E_{HOMO}\left(V\right)=\varepsilon_{h}-\gamma eV$$

where the shift coefficient *γ* of the energy level is provided by

(11) $$\gamma =-\frac{1}{2}\frac{V_{t+}+V_{t-}}{\sqrt{V_{t+}^{2}+10\left|V_{t+}V_{t-}\right|/3+V_{t-}^{2}}}$$

Therefore, for asymmetric junctions, the counterparts of Equations (7) and (8) (also as deduced in [34,41,42]) are as follows:

(12) $$\varepsilon_{h}=2\frac{e\left|V_{t+}+V_{t-}\right|}{\sqrt{V_{t+}^{2}+10\left|V_{t+}V_{t-}\right|/3+V_{t-}^{2}}}$$

and

(13) $$I=GV\frac{\varepsilon_{h}^{2}}{{\left[\epsilon_{h}\left(V\right)\right]}^{2}-{\left(eV/2\right)}^{2}}=GV\frac{\varepsilon_{h}^{2}}{{\left(\epsilon_{h}-\gamma eV\right)}^{2}-{\left(eV/2\right)}^{2}}$$

In accordance with Equation (11), $\left|V_{t^{-}}\right|\neq V_{t^{+}}$ and γ≠0 for asymmetric *I–V* curves, and $\left|V_{t^{-}}\right|\approx V_{t^{+}}$ and *γ* vanishes for symmetric *I–V* curves. Notice that *G* is expressed as Equation (9) identically for both cases.

## 3. Applications of Transition Voltage Spectroscopy in Molecular Junctions

### 3.1. Molecular Transistor with Three-Terminal Electrodes

**Figure 2:**
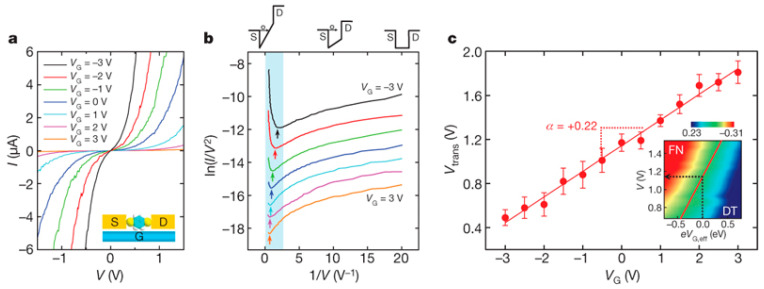
(**a**) Representative *I*–*V* curves of BDT transistors for various values of *V*_G_. (**b**) F–N curves exhibiting the gate-dependent *V_t_*, as indicated by the arrows. (**c**) A relationship between *V_t_* and *V*_G_ with the linear fit (solid line) and the error bars (denote the standard deviation of the individual measurements). Reproduced with permission from [61].

Transistors are archetypally three-terminal electronic devices that control the carrier transport between a source and drain electrode by adjusting the applied gate voltage (*V*_G_). The electrostatic gating of the orbital energy levels in molecular junctions has been suggested in a method similar to conventional field-effect transistors (FETs) [56,57,58,59,60]. The experimental constructions of such a device have been a challenging goal of nanoscale electronics to further shrink devices, as well as provide an analytical platform for exploring charge transport mechanisms at the molecular or atomic scale. Song et al. reported detailed TVS characterization of molecular transistors [61], in which tunneling current was adjusted by the direct modulation of the molecular energy levels in individual molecules with a gate electrode. The transistors were fabricated using an electromigration technique of breaking a very narrow, thin Au wire deposited with the target molecules that were placed onto an oxidized aluminum gate electrode [62,63]. By measuring *V_t_* from *I*–*V* characteristics of benzenedithiol (BDT) molecular transistor at various applied gate voltages (Figure 2a,b), it was observed that a linear relationship occurs between the applied gate voltage and the orbital energy level (Figure 2c). The slope, $\alpha =\Delta V_{t}/\Delta V_{G}$, is specified as the gate efficiency factor, which indicates the amount of orbital energy shift produced by the applied gate voltage. A positive (or negative) gate voltage would lower (or raise) the molecular orbital energy level in respect to *E*_F_, respectively. Thus, the positive slope (i.e., *α* > 0) measured in the BDT transistor clearly shows the characteristics of p-type (hole) transport.

**Figure 3:**
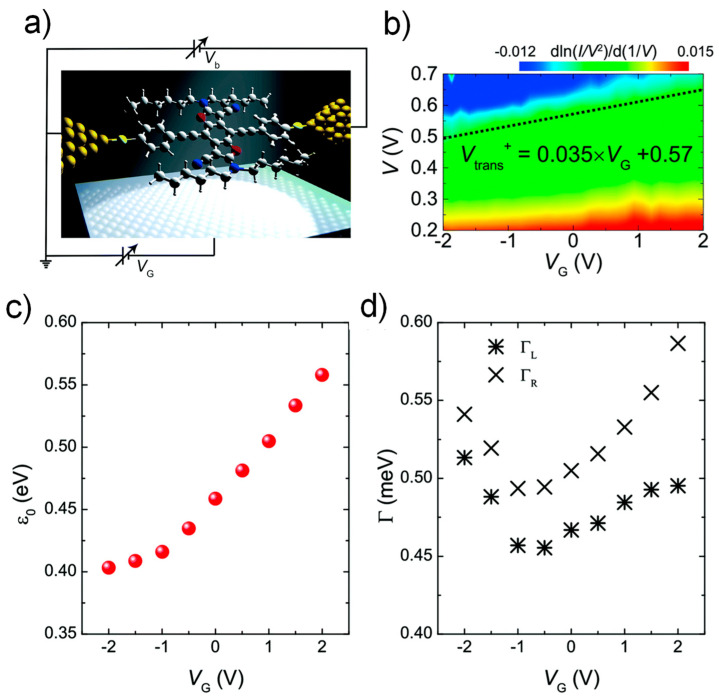
(**a**) Schematic of BDF molecular transistor. (**b**) 2D contour map of *dln*(*I*/*V*^2^)/*d*(1/*V*) versus *V*_G_. *V_t_* when *dln*(*I*/*V*^2^)/*d*(1/*V*) = 0 is indicated by a dashed line. (**c**) The energy offset *ε*_0_ plotted against *V*_G_. (**d**) The gate voltage dependence of the electrode coupling to the left (star) and right (cross) sides. Reproduced with permission from [64].

Xiang et al. examined the effect of molecular orbital gating within the framework of TVS, using benzodifuran (BDF) molecular transistors (Figure 3a) [64]. To apparently display the relationship between the transition voltages and applied gate voltages, two-dimensional contour diagrams of differential value $d\ln\left(I/V^{2}\right)/d\left(1/V\right)$ versus *V*_G_ were displayed in Figure 3b. All the measured values of *V_t_* at the point of $d\ln\left(I/V^{2}\right)/d\left(1/V\right)=0$ were pointed out by the dashed line in Figure 3b. It was shown that *V_t_* is linearly proportional to *V*_G_ with the slope of 0.035. Consequently, HOMO could be confirmed as the closet frontier molecular orbital responsible for the charge transport via the BDF transistor. The dependence of the gate voltage on the orbital level position *ε*_0_ and the contact coupling strength *Γ* was also probed by fitting the *I*–*V* characteristics obtained at various gate voltages employing the Landauer formula (Equation (6)). *ε*_0_ increased monotonically as the gate voltages increased (Figure 3c), indicating that an increase in gate voltage enlarged *ε*_0_ between the HOMO level and *E*_F_. Because the positive potential of *V*_G_ lowers both HOMO and LUMO in respect of *E*_F_, the HOMO is identified to be the dominant transport level in the BDF transistor. Overall, this observation fully accorded with the TVS analysis. In contrast to *ε*_0_, *Γ* appeared to be independent of the gate voltage as seen in Figure 3d, Interestingly, a small change in the strength *Γ* of coupling while sweeping *V*_G_ implies that the applied gate voltage mainly influences the shift of energy levels, but not *Γ* of BDF molecules contacted to Au source and drain electrodes.

### 3.2. Chemical Gating of Molecular Junction Using Edge-on Substituents

**Figure 4:**
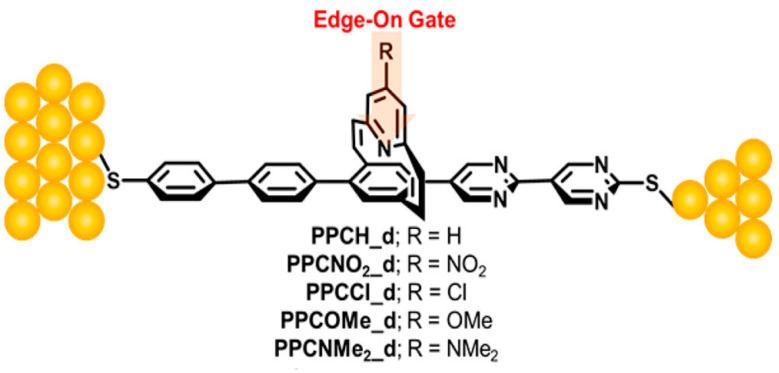
Schematic of a molecular junction with the PPC moiety as a gating part. Reproduced with permission from [66].

**Figure 5:**
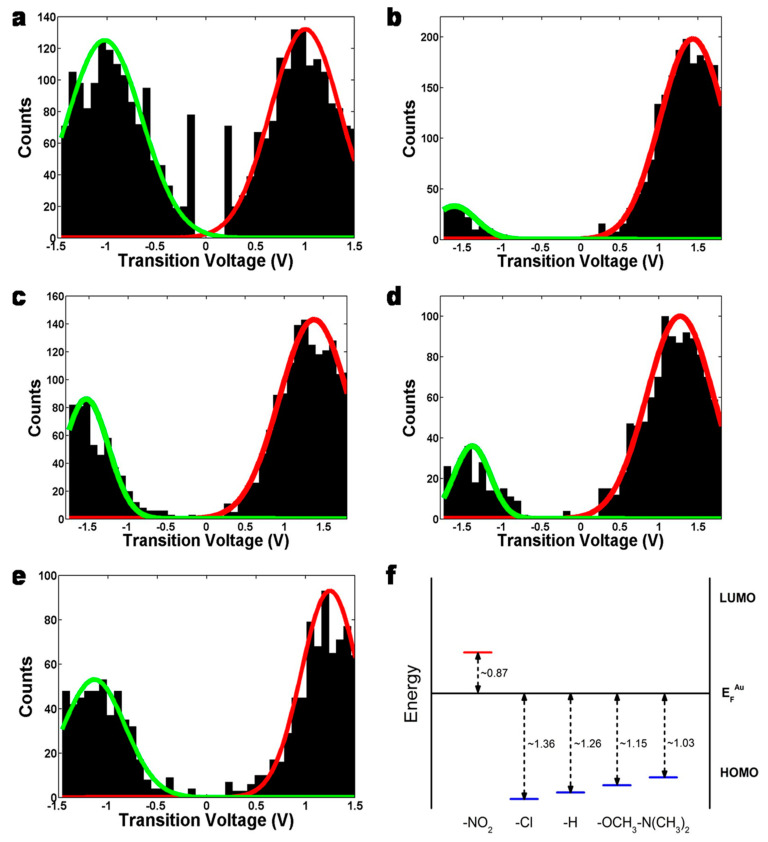
The 1D *V_t_* histograms of (**a**) PPC-NO_2_, (**b**) PPC-Cl, (**c**) PPC-H, (**d**) PPC-OCH_3_, and (**e**) PPC-N(CH_3_)_2_ molecular junctions. (**f**) Energy offsets obtained from the *V_t_* of (**a**–**e**). Reproduced with permission from [65].

Yu et al. recently demonstrated the gating effect by means of edge-on chemical moieties [65,66,67]. They used the pyridinoparacyclophane (PPC) molecules as a gate part in the junctions. In the functional molecular wires, the vertical pyridine moiety was attached together with two vinyl groups to the π-conjugated segment as seen in Figure 4 [66], where the conjugated part in vinyl groups and the pyridine ring was perpendicular to that of the molecular conducting wire. A variety of substituents were connected to the gating site to investigate the effect of the chemical substituents. TVS measurements clearly showed that the molecular orbital levels and then the barrier height for the tunneling transport can be regulated by altering the electronic properties of the gating substituent groups, which were NO_2_, Cl, H, OCH_3_, and N(CH_3_)_2_. 1D histograms of the obtained transition voltages were illustrated in Figure 5a–e [65]. The energy offset between the orbital energy and *E*_F_ can be obtained from Equation (12) (Figure 5f) [65,67], based on TVS analysis as described in Section 2.3. This result apparently demonstrated that as the substituent groups change from electron-donating to electron-accepting moiety, the energy offset was increased consistent with the trend in HOMO-mediated charge transport, except for the PPC molecules with NO_2_ group (Figure 5f). From the DFT calculation, the LUMO of the nitro compound (−3.07 eV) was lowered than other PPCs (around −1.3 ∼ −1.6 eV). Thus, the LUMO was closer to Au electrode’s *E*_F_ for the nitro compound, which indicates that charge transport across the PPC molecular wire with NO_2_ group was indeed tunneling via the LUMO (i.e., n-type transport).

### 3.3. Dependence of Molecular Length and Electrode Work Function

**Figure 6:**
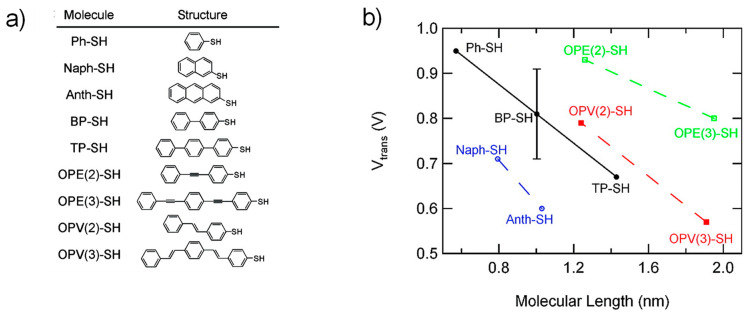
(**a**) The nomenclatures and the molecular chemical structures used in Ref. [32]. (**b**) *V_t_* trend against molecular length for four different molecular types. Reproduced with permission from [32].

It has been well known that the width of the constituent molecules incorporated in the junctions has important effects on both energy level alignments and then the transport characteristics [68,69,70,71,72,73,74,75,76]. Length-variable transport properties of the molecular electronic junctions with π-conjugated aromatic molecules have systemically been examined with TVS [31,32,50]. For example, as displayed in Figure 6, *V_t_* was found to decrease for a series of aromatic thiols as increasing the molecular length [32]. Such consequence can be reasonably anticipated as mediating that the energy difference between the HOMO and LUMO decreases with increasing conjugation lengths for the aromatic molecules [77]. The aromatic molecular junctions examined in this study revealed four different types of conjugation, remarkably displaying a simple reliance upon the length for given molecular series.

**Figure 7:**
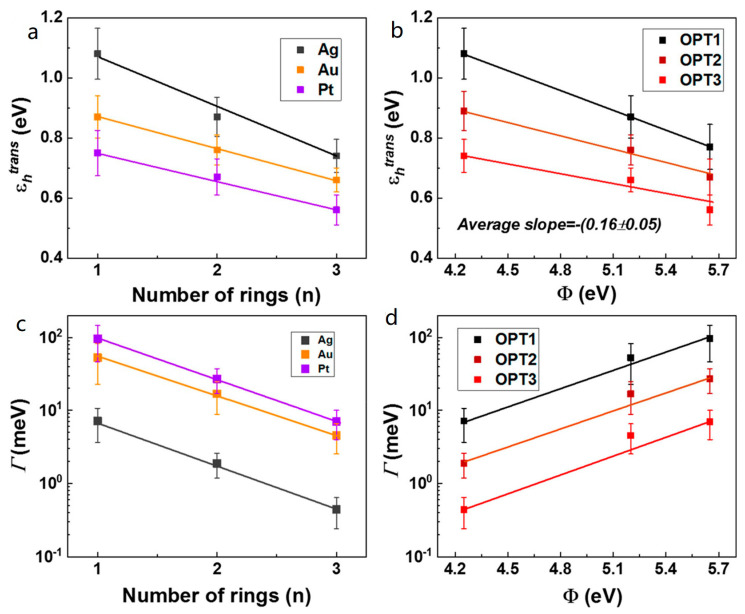
Effective tunneling barrier (or HOMO offset) *ε_h_^trans^* of OPT molecular junctions is displayed as a function of (**a**) molecular length *n* and (**b**) bare electrode work function *Φ*. The dependence of electrode coupling *Γ* of OPT molecular junctions on (**c**) molecular length *n* and (**d**) bare electrode work function *Φ* is demonstrated. Reproduced with permission from [42].

**Figure 8:**
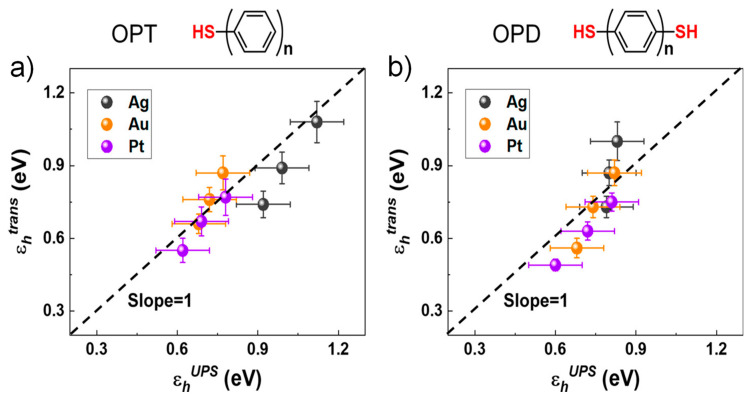
Correlation of *ε_h_^trans^* and *ε_h_^UPS^* obtained from TVS and UPS measurements, respectively, for (**a**) OPT and (**b**) OPD molecular junctions with various electrodes of Ag, Au, and Pt. The dashed lines show the perfect correspondence. Reproduced with permission from [42].

Recently, Xie et al. reported the transport properties of molecular electronic junctions fabricated with the conductive atomic force microscope (CAFM) platform, based on a self-assembled monolayer (SAM) of oligophenylene monothiols (OPT) and dithiols (OPD) on Pt, Au, Ag substrates [42,51]. From fitting of the *I*–*V* data measured from OPT and OPD junctions to the SLM, both *ε_h_* and *Γ* could be obtained in terms of the number of phenyl rings (*n*) and the electrode’s work function (*Φ*). The effective energy offsets *ε_h_^trans^* for OPT molecular junctions were calculated from *V_t_* by using Equation (12), where *ε_h_^trans^* decreased as increasing *n* (Figure 7a). The influence of *ε_h_^trans^* on the work function *Φ* of the bare electrodes was shown in Figure 7b. The result that large changes in *Φ* (more than 1.4 eV) generated somewhat moderate changes (only 0.2 eV) in *ε_h_^trans^* clearly indicated the Fermi level pinning [42]. However, the molecule–electrode couplings *Γ*, calculated by Equation (9), drastically changed with both *n* and *Φ* (Figure 7c,d). It was found that *Γ* of OPT junctions decreased, *Γ*_Ag_ < *Γ*_Au_ < *Γ*_Pt_, also which showed an exponential decay with an increase in *n* for each molecular type. The results described that the conductance and tunneling decay coefficient of oligophenylene-based molecular junctions were largely related to *Γ*, but not *ε_h_*. As shown in Figure 8, independent experiments of *ε_h_^UPS^* measured by ultraviolet photoelectron spectroscopy (UPS) for OPT and OPD SAMs on Pt, Au, and Ag agreed very much with *ε_h_^trans^* estimated from TVS. Because the UPS measurements involved SAMs attached to only one electrode, the correspondence of *ε_h_^trans^* and *ε_h_^UPS^* also shows that the top metal electrode relatively has a gentle effect upon the HOMO energy. A similar TVS study based on SLM approach was also reported for alkyl thiols and dithiols [41], which provided valid verification of hole tunneling transport dominated by localized HOMO states at the contact interface between the Au electrode and thiols, but not by the σ orbital states delocalized in the C–C single bonds. Using TVS, the result clarified a long-standing question about conduction mechanisms of the alkyl-based molecular junctions.

### 3.4. Temperature Dependence

**Figure 9:**
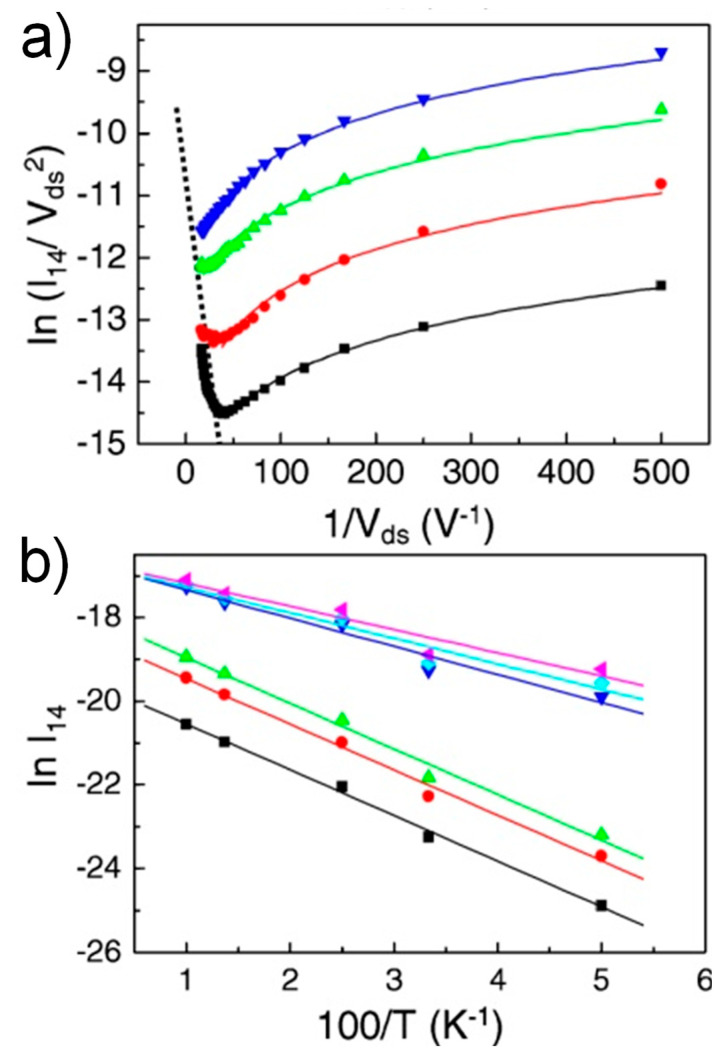
(**a**) F–N plots obtained between 20 K (**bottom**) to 50 K (**top**) with 10 K interval. The dotted line indicates *V_t_*. (**b**) Arrhenius plot of the temperature-dependent *I*–*V* with linear fits (solid lines). The lower three data set was obtained when the drain voltages were 2, 6, and 10 mV (>*V_t_*), respectively. The upper three data set was obtained when the drain voltages are 50, 54, and 58 mV (<*V_t_*), respectively. Reproduced with permission from [83].

In order to clearly understand a conduction mechanism in molecular electronic junctions, it is necessary to study the temperature dependence of charge transport behavior [78,79,80,81,82]. Chiu et al. performed temperature-variable TVS analysis of metal-semiconductor carbon nanotube heterojunctions at a large gate voltage, in which the effect of Schottky barrier on charge transport can be very weak [83]. The F–N plots were shown against temperature varying from 20 to 50 K (Figure 9a). A change in tunneling mechanism from direct tunneling to field emission was seen at 20 K. At the low bias regime, where direct tunneling occurs, the F–N plots at all temperatures showed the logarithmic growth but shifted up as temperature increased. *V_t_* shifted to greater voltage as temperature increased, which was ascribed to the thermally activated process (Figure 9b). Specifically, charge carriers can surpass the potential barrier via thermionic emission with increasing temperature, then that which contends with field emission at *V_t_*. After all, the voltage required to give rise to the field emission was higher (to make the barrier thinner), and the transition was gradually smeared out for the range of measured voltages with increasing temperature. These experimental results indicated that the barrier of charge transport at the metal-nanotube interface could be probably very low with the high gate bias.

**Figure 10:**
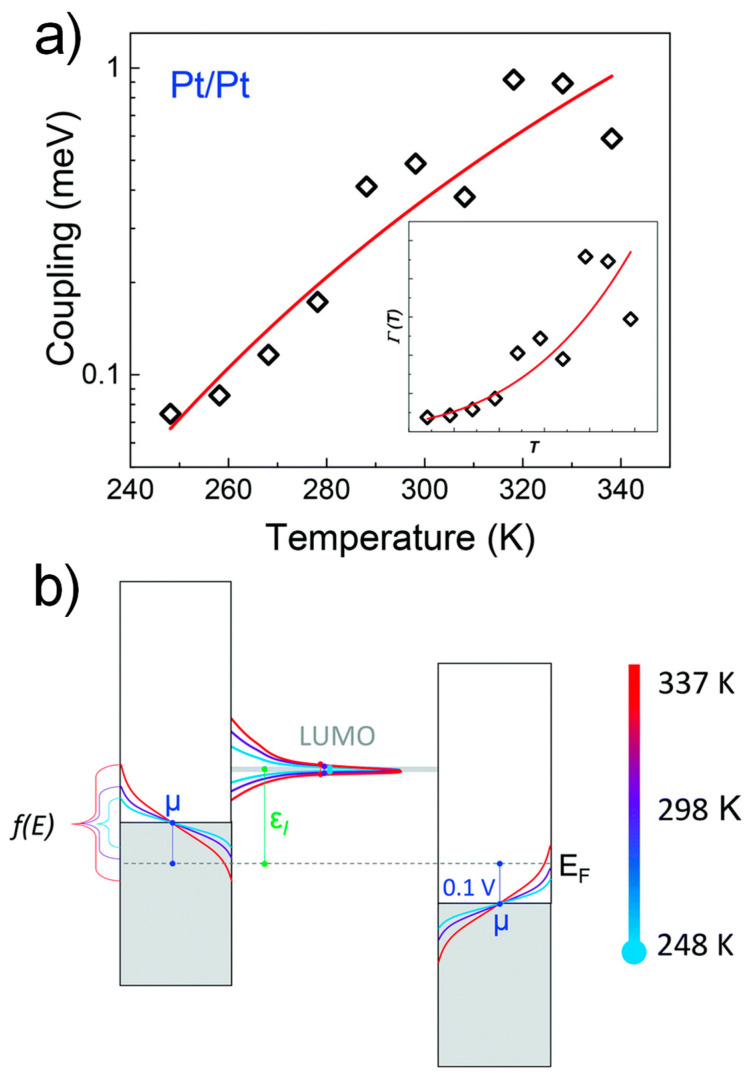
(**a**) Semi-log plot of the effective coupling *Γ*(*T*) extracted from the variable temperature *I*–*V* of the PDI molecular junction. The inset: the same data in a linear scale. (**b**) A qualitative energy diagram of the PDI molecular junction with the Fermi distribution of the contacts. The electrode coupling *Γ*(*T*) of the LUMO is at 0.2 V. Reproduced with permission from [52].

Recently, Smith et al. showed that the temperature dependence of charge transport for the isocyanide perylene diimide (PDI) molecular junctions originated from the strong increase of the effective molecule–electrode coupling *Γ*(*T*) with an increase in temperature using the SLM analysis combined with TVS measurements (Figure 10a) [52]. This was most likely because of the thermal broadening of the charge carrier’s energy distributions (Figure 10b) and not the coupling to vibrational (phonon) modes in the electrodes [84].

### 3.5. Ambipolar Transition Voltage Spectroscopy

**Figure 11:**
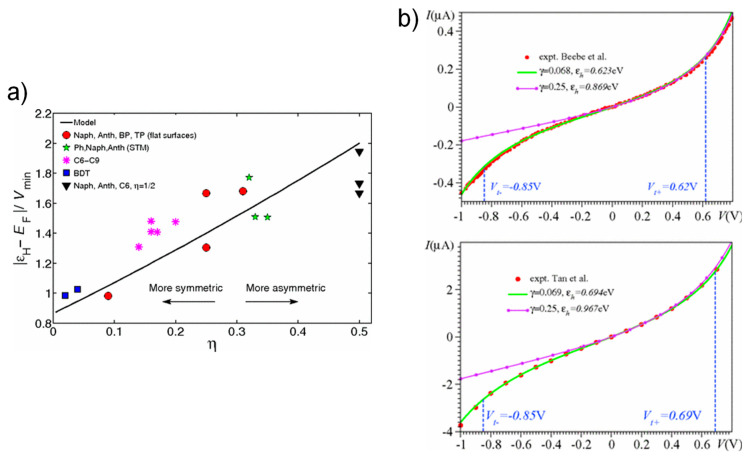
(**a**) Ratio of the HOMO level at zero bias to *V_t_* as a function of the asymmetry parameter. The solid line is calculated by a Lorentzian transmission function (Equation (5)). Reproduced with permission from [44]. (**b**) The *I*–*V* curves of anthracene and terphenyl-based junctions measured with CAFM setup by Beebe et al. (**upper panel**) [31] and Tan et al. (**lower panel**) [36], respectively. The solid lines are the theoretical curves via Equation (13) in conjunction with the *ε_h_* and *Γ* obtained from the TVS measurement and the value *Γ* = 0.25 from the DFT calculation [44]. The theoretical curves of each panel disagree with experiments at negative voltages. Reproduced with permission from [34].

Chen et al. reported that the proportion of *V_t_* to the HOMO position *ε_h_* varied between 0.85 (in the case of symmetric junctions) and 2.1 (in the case of fully asymmetric junctions) according to the contact asymmetry (*η*) of molecular electronic junctions [44], as illustrated in Figure 11a. Such large variation results from a discrepancy in the nonlinear response of the orbital energy level to the applied voltage. In Figure 11a, the plotted line was the outcome acquired from the Lorentzian transmission function (Equation (5)) and the Landauer formula (Equation (6)); the symbols were obtained from calculations of ab initio finite bias. Wang et al. also reported consistent experimental investigation into the effect of the asymmetric coupling on the TVS analysis [85]. The results from these studies apparently followed the same propensity, implying that it is inevitable to deliberate the asymmetry to employ TVS as a quantitative analytic technique for probing the electronic structure in molecular electronic junctions.

Later, Bâldea et al. suggested ambipolar TVS derived from polarity-dependent transition voltages [34], in which the transition voltages *V_t_*_±_ for the polarities of positive and negative bias can be employed to accurately establish the energy offset of the orbital energy level nearest to *E*_F_, as well as the degree of junction’s bias asymmetry. Using the ambipolar TVS technique, the full *I*–*V* characteristics experimentally obtained by Ref. [31] and Ref. [86] could be excellently reproduced for both bias polarities (Figure 11b). This result showed that TVS was properly used to determine both the molecular orbital level position and the voltage division factor *γ* in the junctions. To this end, only quantities that should be estimated experimentally are the *V_t_*_±_ for both bias polarities. Noticeably, the *Γ* value was considerably smaller than that determined by DFT calculations [44], implying that there is a limit of the DFT-based approaches to solve charge transport problems for molecular junctions.

### 3.6. Molecule–Electrode Interface

It has been well recognized that the interfacial properties between the constituent molecules and contact electrodes significantly affect the charge transport through molecular junctions [87,88,89,90,91,92,93,94,95]. A variety of experimental studies demonstrated that the TVS technique can be utilized to investigate diverse molecule–electrode interfacial issues involved in the molecular junctions. For instance, *V_t_* decreased for hole tunneling transport via HOMO with the thiol end-group as increasing electrode’s work functions, but it changed in the opposite direction for electron tunneling transport via LUMO with the isocyanide end-group [96]. The degree of a change in *V_t_* was significantly smaller as compared to that of the work functions. The observation showed that a variety of specific bonding sites of the terminal groups with varied electrodes can affect the measured *V_t_* values. Bennett et al. showed a multipeak in the statistical distribution of *V_t_* for the porphyrin molecular wires with thiol linkages [88], originating from various bonding site configurations existing at the molecule–electrode contacts. The gap size between the contact electrodes and the component molecules has a significant influence over *V_t_*, because different orbital levels can take part in charge transport through the junctions depending on the molecule–electrode distance [89]. It was also discovered that a smaller value of *V_t_* can be incurred by the surface oxidized in Si, GaIn, or Hg electrodes [90]. Guo et al. reported the robust statistical TVS study based on plenty of experimental *I–V* data. They investigated transition voltage spectra of the alkanethiol junctions with distinct contact interfacial configurations employing the single-molecule break junction technique [97], where the *V_t_* values were similar (∼1.4 V) notwithstanding a large variation in the junction conductance measured with different molecular lengths. This was attributed to a difference in contact resistance (or molecule–electrode coupling strength) but not variation in the orbital level position, indicating that the coupling strength was the major origin of observed conductance distribution in the alkyl-based molecular junctions. Recently, Ge et al. described by using TVS measurement how the surface d-bands in transition metal electrodes consolidate the molecular adsorption and then enhance interfacial charge transport [98]. This result provided a new method to optimize molecular electronic devices with transition metal electrodes.

## 4. Conclusions

As discussed in this review, TVS should be a useful analytical tool particularly to investigate coherent non-resonant tunneling transport extensively observed in molecular electronic junctions [99,100,101,102,103,104]. The SLM-based TVS approach has so far been a valuable framework for quantitative characterization of charge transport through these junctions. The recent experimental studies showed that TVS can present important information on the energy level alignment, including the energy offset, the dominant transport orbital, the molecule–electrode coupling, the Fermi level pinning, the molecular orbital gating, the interfacial property, etc. Overall, the analyses described in this review provide an improved comprehension of the relationship between the energy level alignment and charge transport mechanisms. It is of essential importance for the development of new molecular devices with desired functionalities.

## Data Availability

Not applicable.
